# Features of a FAIR vocabulary

**DOI:** 10.1186/s13326-023-00286-8

**Published:** 2023-06-01

**Authors:** Fuqi Xu, Nick Juty, Carole Goble, Simon Jupp, Helen Parkinson, Mélanie Courtot

**Affiliations:** 1 European Bioinformatics Institute (EMBL-EBI), European Molecular Biology Laboratory, Wellcome Genome Campus, Cambridge, Hinxton CB10 1SD UK; 2 The University of Manchester, Oxford Rd, Manchester, M13 9PL UK; 3 SciBite BioData Innovation Centre, Wellcome Genome Campus, Hinxton, CB10 1DR UK; 4 Ontario Institute for Cancer Research, 661 University Ave Suite 510, Toronto, M5G 0A3 Canada; 5 Department of Medical Biophysics, University of Toronto, Toronto, ON M5G 1L7 Canada

**Keywords:** FAIR principles, Ontology, Vocabulary, FAIR assessment

## Abstract

**Background:**

The Findable, Accessible, Interoperable and Reusable(FAIR) Principles explicitly require the use of FAIR vocabularies, but what precisely constitutes a FAIR vocabulary remains unclear. Being able to define FAIR vocabularies, identify features of FAIR vocabularies, and provide assessment approaches against the features can guide the development of vocabularies.

**Results:**

We differentiate data, data resources and vocabularies used for FAIR, examine the application of the FAIR Principles to vocabularies, align their requirements with the Open Biomedical Ontologies principles, and propose FAIR Vocabulary Features. We also design assessment approaches for FAIR vocabularies by mapping the FVFs with existing FAIR assessment indicators. Finally, we demonstrate how they can be used for evaluating and improving vocabularies using exemplary biomedical vocabularies.

**Conclusions:**

Our work proposes features of FAIR vocabularies and corresponding indicators for assessing the FAIR levels of different types of vocabularies, identifies use cases for vocabulary engineers, and guides the evolution of vocabularies.

**Supplementary Information:**

The online version contains supplementary material available at 10.1186/s13326-023-00286-8.

## Background

The Findable, Accessible, Interoperable and Reusable (FAIR) Principles [1] have rapidly gained traction in the biomedical community since their publication in 2016, with many groups attempting to improve their data quality, develop FAIR capable data resources, and design generic FAIR assessment tools for biomedical data [2–4]. The heterogeneous nature and broad scope of biomedical data, ranging from molecular data to human studies via interdisciplinary analysis, stringent requirements for data FAIRness can ensure the usefulness of such data in benefiting human health. While assessing the FAIR level of datasets and data resources [5], we noted a recursive cycle with respect to the ‘Interoperable’ FAIR Principle, *“I2 - (Meta)data use vocabularies that follow FAIR principles”*. To comply with that principle, datasets need to use FAIR vocabularies, which themselves need to be FAIR. However, it remains unclear what is a FAIR vocabulary. Having FAIR vocabularies promote biomedical data FAIRness throughout the data life cycle, during the data generation, curation, and distribution processes, and support data exchange and integration across data resources. Therefore, it is crucial to clarify the definition and features of a FAIR vocabulary.

Standards for FAIR vocabularies have been generated in different domains. The FAIRsFAIR recommendations [6] provide guidance on FAIR semantic artefacts, as well as supporting vocabulary search engines and repositories. Garijo and Poveda-Villalon [7] discussed detailed requirements of ontology, such as Uniform Resource Identifiers(URI), versioning strategies, and the formatting of those ontologies. Furthermore, “Ten simple rules for making a vocabulary FAIR [8]” for converting print-based or other forms of legacy vocabularies to FAIR vocabularies have also been proposed.

Researchers have also developed indicators and services to assess the FAIR level of digital objects both manually and automatically; FAIR indicators in the FAIRsharing [9] FAIR Maturity Evaluation Service [4], FAIR metrics in the F-UJI Automated FAIR Data Assessment Tool [10], and the Research Data Alliance (RDA) Data Maturity Model Specification and Guidelines [11], also known as the RDA indicators. Among them, the RDA indicators are a set of representative and descriptive indicators to evaluate the FAIR level of data which have been used in many projects and with diverse data types [5]. Some automated assessment services, such as FOOPS! [12], have been developed to measure the FAIR level of public, machine-readable vocabularies. The Open Biomedical Ontology(OBO) Dashboard service checks how ontology adheres to OBO principles [13].

Vocabularies come in different forms, such as lists, thesaurus, taxonomies, and ontologies; each at different levels of semantic complexity and FAIRness and can all archive various levels of FAIR. To the best of our knowledge, there have not yet been quantifiable FAIR assessment approaches developed to measure the FAIR level of different formats of vocabularies objectively.

Therefore, in this paper, we distinguished the concepts of FAIR data, FAIR data resources, and FAIR vocabularies, and propose a set of general FAIR Vocabulary Features (FVFs) as a set of satisfiable features for vocabularies. We also adapted the RDA indicators to measure the FAIR levels of vocabularies. Further, we provided example assessments based on selected ontologies available from the EMBL-EBI Ontology Lookup Service(OLS) [14] and other vocabulary resources.

## Results

### Defining FAIR Data and FAIR vocabulary

To describe the features of a FAIR vocabulary, the first step is to define what a FAIR vocabulary is, and how to distinguish it from FAIR data. In our analysis, data can be FAIR, to a greater or lesser extent, and data resources and data vocabularies are capable of supporting FAIRness at different levels. Data vocabularies are designed to support FAIR data, and they can also be considered FAIR data resources. The orthogonality of these concepts is an important context for this work when determining the features of a FAIR vocabulary. Table 1 presents a definition for FAIR data, FAIR data resources, and FAIR vocabulary.

**Table 1:** Definitions of FAIR data, FAIR vocabularies and FAIR metadata

Concept	Definition
FAIR Data	FAIR data are data which have been subjected to some assessment process and for which some resulting evaluation of FAIRness is available.
FAIR capable resource	A FAIR capable data resource is a data resource which has been subjected to some assessment process and for which some resulting evaluation of FAIR capability is available.
FAIR Vocabulary	A vocabulary which is determined to be FAIR by assessment of the vocabulary itself and its use in the delivery of FAIR data.

A FAIR vocabulary has a set of FAIR features and also has a list of associated FAIR indicators. It is usable for annotation, analysis and presentation of data, and is deployable in the context of a FAIR-capable data resource or tools. It also serves ‘aggregation’ use cases where data originates from different domains and enables data interoperability, such as concept mapping, where different vocabularies are used. Requirements for a FAIR vocabulary cover three aspects:

1. FAIR in terms of its application to FAIR data
2. FAIR in the context of FAIR capable resources
3. FAIR in the context of other vocabularies

### Alignment with existing standards

We analysed the possibility of reusing the OBO principles to define the FAIR vocabulary features. We noted that not all the OBO principles are expressed at the same level of maturity or granularity, and therefore some were unmappable and excluded from the features. Whilst the FAIR principles apply to both ontology developers and ontologies themselves, only principles that focus on ontologies as digital objects were selected. As a result, this analysis did not include *OBO Principle 1: Open*, *4: Versioning*, *6: Textual definitions*, *9: Documented plurality of users*, *10: Collaboration commitment*, *11: Authority*, *12: Naming conventions*, and *20: responsiveness*. The rationale for suitability as FVFs is discussed in detail in Supplementary Table 1.

### FAIR vocabulary features

Based on the analysis of the OBO foundry practices, FAIR principles, and our previous experience working with and developing ontologies, we proposed eleven features for FAIR vocabulary in Table 2, covering requirements for identifiers, access protocols, knowledge representation, and other aspects. The relationships among FVF, the FAIR principles and three aspects of FAIR vocabularies are presented in Tabel 3. The FVFs cover all four aspects of the FAIR principles with a focus on the interoperability aspects of FAIR data and data resources.

**Table 2:** FAIR Vocabulary Feature details

ID	Features	Description	Examples
FVF-1	Vocabulary and constituent terms are assigned globally unique and persistent identifiers.	Vocabulary and constituent terms should have identifiers that are globally unique and persistent to ensure that each item can be identified unambiguously over time.	Examples of globally unique and persistent identifiers are PURL [15], identifiers.org [16], and w3id.org [17]. The OBO foundry provides an identifier policy [18] for biomedical ontologies and requires the use of PURLs with standard prefixes, such as http://purl.obolibrary.org/obo/GO_0000022.
FVF-2	Vocabulary and constituent terms have rich metadata.	Vocabulary and constituent terms should have sufficient metadata to support discovery by both humans and machines.	Vocabulary metadata should provide information about the creation date, creator and editor, version, licence, target domain and short description. Metadata should describe term editing history, definition source, and other metadata.
FVF-3	Vocabulary and constituent terms can be accessed using identifiers, preferably by both humans and machines.	The URIs for the vocabulary itself and its constituent terms can be dereferenced by both humans and machines.	http://www.ebi.ac.uk/efo/EFO_0000311 resolves to the Term “Cancer” in the Experimental Factor Ontology(EFO), which can be accessed by both humans using ontology browsers and machines through the OLS API.
FVF-4	Vocabulary and constituent terms are registered or indexed in a searchable engine or a resource.	The vocabulary itself and its constituent terms are registered in vocabulary archives or other vocabulary management systems and are indexed by local or/and global search engines.	EMBL-EBI Ontology Lookup Service(OLS) and NCBI BioPortal [19]are two popular public vocabulary archives. Property *X-Robots-Tag:index* in vocabularies allows them to be indexed by search engines.
FVF-5	Vocabulary and constituent terms are retrievable using a standardised communication protocol, preferably open, free and universally implementable protocols, which allow for authentication and authorisation, where necessary.	The vocabulary itself and its constituent terms are retrievable using a standardised communications protocol, preferably open, free and universally implementable protocols, such as HTTPS, HTTP or FTP. The protocol should also allow identification of the user and grant access based on their associated privilege, when necessary.	Most public ontologies can be accessed using HTTP or HTTPS protocols. For example, EFO uses HTTP, while the Unified Medical Language System [20] uses the HTTPS protocol, only allowing access to authenticated users.
FVF-6	Vocabulary and constituent terms are persistent over time and are appropriately versioned.	Changes in the vocabulary are reflected in different versions. Vocabularies and their terms are versioned, and each unaltered version of the vocabulary can be identified and retrieved in perpetuity. Vocabulary metadata is available even when the vocabulary is no longer available.	Changes in EFO are included in each release and identified with versioned IRI, such as http://www.ebi.ac.uk/efo/releases/v3.31.0/efo.owl, which resolves to the versioned vocabulary. OBO Foundry also provides guidelines [21] for ontology versioning and how different versions of the vocabularies should be labelled, stored and published.
FVF-7	Vocabulary and constituent terms use a formal, accessible and broadly applicable, and preferably machine-understandable language for knowledge representation.	The vocabulary itself and its constituent terms use a formal, accessible and broadly applicable, and preferably machine-understandable language for knowledge representation.	OWL-based vocabularies can be serialised using RDF-XML, or relational databases e.g. ChEBI [22] can be converted into OWL [23]
FVF-8	Vocabulary and constituent terms use qualified references to other vocabularies.	Vocabulary reuses terms from other vocabularies when applicable, provides adequate metadata about external terms, and follows vocabulary cross-reference standards.	EFO reuses human anatomy terms such as “liver” from UBERON [24](UBERON_0002107) and links to the original UBERON term. Property *Xref* indicates a cross-reference relationship between two vocabulary terms. MIREOT [25] defines a methodology and minimum information requirements for importing external terms into an extant ontology.
FVF-9	Vocabulary and constituent terms are described with a plurality of accurate and relevant attributes.	Vocabulary terms include sufficient attributes, such as labels, synonyms, definitions, examples of usage, and cross-references, to support the interpretation and reuse of vocabulary terms.	The OBO flat-file format specification [26], synonym, Xref, relationship, etc. The Minimal requirement for term annotations in OBI (metadata) [27] also specifies minimum requirements for each ontology term.
FVF-10	Vocabularies are released with a standard data usage licence, preferably a machine-readable licence.	The vocabulary includes information about how the vocabulary can be reused.	Common public data usage licences are CC-BY [28] and MIT [29]. For example, Gene Ontology uses Creative Commons Attribution 4.0 Unported License. SNOMED^TM ^[30]uses a self-defined SNOMED CT^TM^ affiliate license agreement.
FVF-11	Vocabularies meet domain-relevant community standards.	Vocabularies cover essential terms for the specific domain, reflect knowledge of this domain and can be used in existing data standards and data models.	Community standards, such as minimum information requirements and data models can be found in FAIRsharing [9]. The Plant Phenotyping Experiment Ontology(PPEO) [31] implements the Minimum Information about Plant Phenotyping Experiment(MIAPPE) [32] standards and covers essential attributes to describe a MIAPPE-compliant phenotype dataset.

Table 2 also provides examples for each FAIR feature representing in different formats and at varying FAIRness levels amongst those vocabularies. For example, for *FVF-6: versioning and persistent vocabularies*, of all ontologies indexed and updated in OLS, 59.3$\%$ of selected vocabularies use a date format of *“yyyy-mm-dd”* in the *“versionIRI”*, such as http://purl.obolibrary.org/obo/scdo/releases/2021-04-15/scdo.owl. 2.5$\%$ of vocabularies use semantic versioning, such as http://www.ebi.ac.uk/efo/releases/v3.34.0/efo.owl or other forms of numeric versioning, such as http://www.orpha.net/version3.2. 31.7$\%$ of vocabularies do not provide valid machine-readable versioned IRIs. For *FVF-1: identifiers*, 74$\%$ of vocabularies use OBO-format Persistent Uniform Resource Locators (PURL), identifier.org, w3id.org identifiers, as well as other domain-specific identifiers. For *FVF-5: accessible using standard protocols*, of all 199 selected ontologies, only one ontology uses the HTTPS protocol; the rest use HTTP protocols.

**Table 3:** FAIR vocabulary features mapped to FAIR principles and FAIR vocabulary requirements

Aspects of FAIR vocabulary	Findability	Accessibility	Interoperability	Reusability
FAIR in terms of application to FAIR data.			FVF-11	
			FVF-2	
			FVF-6	
			FVF-9	
			FVF-10	
			FVF-11	
FAIR in terms of serving as a FAIR data resource.	FVF-1	FVF-3	FVF-7	FVF-2
	FVF-4	FVF-5		FVF-6
	FVF-6	FVF-10		FVF-7
				FVF-9
FAIR in the context of interacting with other vocabularies.			FVF-8	

### Indicators for FAIR vocabulary features

While FVFs identify general characteristics of a FAIR vocabulary, these features need to be objectively quantified to be useful in vocabulary selection, development and assessment. Hence, we aligned FVFs with FAIR indicators to enable the computation of a discrete FAIR score.

We mapped the RDA indicators to FVF, filtering out indicators that do not apply to vocabularies. For example, *RDA-F3-01M: Metadata includes the identifier for the data* is not applicable to ontologies and other types of vocabularies, where the metadata is usually directly embedded within the vocabulary data. We also specified the digital object to which the indicator refers, and identified within each indicator the relevant standards used in corresponding domains. The FVFs, associated with selected indicators, can be used as indicators for FAIR Vocabulary as shown in Table 4. Other indicators that are not suitable for FAIR vocabularies are listed in Supplementary Table 2.

**Table 4:** Indicators for FAIR vocabulary features. Alignment between the FAIR vocabulary features and RDA data maturity level indicators

**FAIR vocabulary Feature**	**RDA indicator ID**	**Indicator**
FVF-1: Vocabulary and their terms are assigned globally unique and persistent identifiers.	RDA-F1-01M	Metadata is identified by a persistent identifier
	RDA-F1-01D	Data is identified by a persistent identifier
	RDA-F1-02M	Metadata is identified by a globally unique identifier
	RDA-F1-02D	Data is identified by a globally unique identifier
FVF-2: Vocabularies and their terms have rich metadata.	RDA-F2-01M	Rich metadata is provided to allow discovery
FVF-3: Vocabularies and their terms can be accessed using the identifiers, preferably by both humans and machines.	RDA-A1-01M	Metadata contains information to enable the user to get access to the data
	RDA-A1-02M	Metadata can be accessed manually(i.e. with human intervention)
	RDA-A1-02D	Data can be accessed manually(i.e. with human intervention)
	RDA-A1-03M	Metadata identifier resolves to a metadata record
	RDA-A1-03D	Data identifier resolves to a digital object
	RDA-A1-05D	Data can be accessed automatically(i.e. by a computer program)
FVF-4: Vocabularies and their terms are registered or indexed in a searchable engine or a resource.	RDA-F4-01M	Metadata is offered in such a way that it can be harvested and indexed
FVF-5: Vocabularies and their terms are retrievable using a standardised communications protocol, preferably open, free and universally implementable protocols. and allows for authentication and authorisation, where necessary.	RDA-A1-04M	Metadata is accessed through standardised protocol
	RDA-A1-04D	Data is accessible through standardised protocol
	RDA-A1.1-01M	Metadata is accessible through a free access protocol
	RDA-A1.1-01D	Data is accessible through a free access protocol
	RDA-A1.2-01D	Data is accessible through an access protocol that supports authentication and authorisation
FVF-6: Vocabularies and their terms are persistent over time and are appropriately versioned.	RDA-A2-01M	Metadata is guaranteed to remain available after data is no longer available
	RDA-R1.2-01M	Metadata includes provenance information according to community-specific standards
	RDA-R1.2-02M	Metadata includes provenance information according to a cross-community language
FVF-7: Vocabularies and their terms use a formal, accessible and broadly applicable, and preferably machine-understandable language for knowledge representation.	RDA-I1-01M	Metadata uses knowledge representation expressed in standardised format
	RDA-I1-01D	Data uses knowledge representation expressed in standardised format
	RDA-I1-02M	Metadata uses machine-understandable knowledge representation
	RDA-I1-02D	Data uses machine-understandable knowledge representation
FVF-8: Vocabularies and terms use qualified references to other vocabularies.	RDA-I3-02D	Data includes qualified references to other data
	RDA-I3-03M	Metadata includes qualified references to other metadata
FVF-9: Vocabularies and terms are described with a plurality of accurate and relevant attributes.	RDA-R1-01M	Plurality of accurate and relevant attributes are provided to allow reuse
FVF-10: Vocabularies are released with a standard data usage licence, preferably a machine-readable licence.	RDA-R1.1-01M	Metadata includes information about the licence under which the data can be reused
	RDA-R1.1-02M	Metadata refers to a standard reuse licence
	RDA-R1.1-03M	Metadata refers to a machine-understandable reuse licence
FVF-11: Vocabularies meet domain relevant community standards.	RDA-R1.3-01M	Metadata complies with a community standard
	RDA-R1.3-01D	Data complies with a community standard
	RDA-R1.3-02M	Metadata is expressed in compliance with a machine-understandable community standard
	RDA-R1.3-02D	Data is expressed in compliance with a machine-understandable community standard

### FAIR assessments for common data vocabularies

We tested the FVF indicators in three representative ontologies. Both the Gene Ontology (GO) and Experimental Factor Ontology (EFO) are vocabularies of high FAIR level, with over 80% FVFs fulfilled (See details in Table 5). GO only partially complies with ‘*FVF-6: Vocabularies and their terms are persistent over time and are appropriately versioned*‘, with a *Fail* in ‘*Indicator RDA-R1.2-02M: Metadata includes provenance information according to a cross-community language*‘. ‘*FVF-2: Vocabularies and their terms have rich metadata*’ was not complied with since no general description of the ontology is provided in the released artefact. Compared with these two ontologies, the taxonomy, International Classification of Diseases 11th Revision (ICD-11) [33], fully complies with 18.18% FVFs and partially complies with 36.36% FVFs. This is because ICD-11 neither refers to other vocabularies within each term description or within the metadata for those terms nor adheres to other community standards, such as vocabulary formats. ICD-11 was selected for this evaluation as it already offers significant FAIR improvements over ICD-10 [34], such as providing a standard licence.

**Table 5:** FAIR vocabulary feature applied. Assessment results of Gene ontology, Experimental factor Ontology and ICD-11

FAIR vocabulary Feature	Vocabulary		
	Gene Ontology	Experimental Factor Ontology	ICD-11
FVF-1: Vocabulary and their terms are assigned globally unique and persistent identifiers.	Full Compliance	Full Compliance	Partial Compliance
FVF-2: Vocabularies and their terms have rich metadata.	Full Compliance	No Compliance	Full Compliance
FVF-3: Vocabularies and their terms can be accessed using the identifiers, preferably by both humans and machines.	Full Compliance	Full Compliance	Partial Compliance
FVF-4: Vocabularies and their terms are registered or indexed in a searchable engine or a resource.	Full Compliance	Full Compliance	No Compliance
FVF-5: Vocabularies and their terms are retrievable using a standardised communications protocol, preferably open, free and universally implementable protocols. and allows for authentication and authorisation, where necessary.	Full Compliance	Full Compliance	Full Compliance
FVF-6: Vocabularies and their terms are persistent over time and are appropriately versioned.	Partial Compliance	Partial Compliance	Partial Compliance
FVF-7: Vocabularies and their terms use a formal, accessible and broadly applicable, and preferably machine-understandable language for knowledge representation.	Full Compliance	Full Compliance	No Compliance
FVF-8: Vocabularies and terms use qualified references to other vocabularies.	Full Compliance	Full Compliance	Partial Compliance
FVF-9: Vocabularies and terms are described with a plurality of accurate and relevant attributes.	Full Compliance	Full Compliance	No Compliance
FVF-10: Vocabularies are released with a standard data usage licence, preferably a machine-readable licence.	Full Compliance	Full Compliance	Full Compliance
FVF-11: Vocabularies meet domain relevant community standards.	Full Compliance	Full Compliance	No Compliance
**FAIR Vocabulary Feature summary**			
FVF, full compliance	90.91$\%$	81.82$\%$	27.27$\%$
FVF, partial compliance	9.09$\%$	9.09$\%$	36.36$\%$
FVF, no compliance	0.00$\%$	9.09$\%$	36.36$\%$

## Discussion

Comparing the assessment results of the two ontologies, GO and EFO, with the list-type dictionary, ICD-11, ontology-based vocabularies follow stricter semantics and therefore fared better in the scoring of FAIR features. One of the reasons is that many ontology-related standards have been established, including formats, such as the Web Ontology Language (OWL), guidelines such as the OBO principles, minimum information standards, such as Minimum Information for Biological and Biomedical Investigations (MIBBI) [35], and mechanisms for cross-references or incorporating external ontologies, such as the Minimum Information to Reference an External Ontology Term (MIREOT) [25]. This is naturally reflected in a higher score for compliance with community standards, which is a core part of FVF, and which improves the interoperability and reusability of a vocabulary. However, being a FAIR ontology does not ensure the quality and usability of an ontology. The scope, popularity, and accuracy of a vocabulary are also factors to consider.

The FVFs we proposed integrate multiple FAIR vocabulary requirements and serve as FAIR vocabulary standards to guide the development and maintenance of vocabularies. Each FVF is associated with indicators, to support its quantifiable and objective assessment against each feature. These indicators can also be plugged into existing or emerging standards in other domains to support the evolving of new vocabularies and suit emerging use cases. For example, in *FVF-8: cross-referencing *other vocabularies can be linked to the ontology cross-reference standards, such as MIREOT. Because of our expertise and requirements, this manuscript focuses on the biomedical domain; however, we anticipate this framework could be reused elsewhere.

Compared with other FAIR vocabulary requirements, FVFs apply to multiple vocabulary formats, and we demonstrated the potential for using them across other forms of vocabularies with the ICD-11 example. We focused on how FVFs can be applied to ontologies and did not include other types of vocabulary specifications, because an ontology has a clearly defined structure, schema, standards, repositories, and supporting standards.

Integrating the FVF with FAIR indicators makes it possible to assess the FAIR level of vocabularies, identify progressive ontology development use cases, and improve accordingly. We selected the RDA indicators since they have proven to be useful in many datasets, and have been referenced by other assessment approaches in FAIRassist.org; yet, FVFs could alternatively be aligned to other FAIR-principle-based indicators which would similarly reflect the FAIR principles. The RDA indicators are designed to evaluate biomedical datasets, where data refers to outcomes of sequencing or screening experiments, and metadata refers to sample information, experiment designs, etc, which needs to be annotated with controlled vocabularies. In our context, data refers to the vocabulary themselves, and metadata, on the other hand, points to ontology versioning and editing information. When performing an assessment, it is crucial for assessors to agree on the definition of data and metadata.

Besides manual assessments, quantifiable formal indicators are also amenable to becoming machine-actionable. Reusing shared indicators will make it possible to perform automated FAIR vocabulary assessments. The bottleneck of automated assessment, however, is the variations in the implementation of the same requirement. For example, the VersionIRI case presented above demonstrates the challenges of exhausting all formats of interpretation to build a unified assessment service. Other features, such as “Complying with domain standards” are even harder to automate. Therefore, manual assessment using indicators for FVFs is still one of the more practical and accurate approaches.

The FAIR scores provide a quantitive and intuitive “summary” of the FAIR level of a vocabulary and can be an effective measure of how the vocabulary has evolved. However, it should neither be taken as an absolute measure to evaluate either the quality comparison across vocabularies or compare different vocabularies. For example, for vocabularies which are used and shared within an institution and not designed for external usage, *having global identifiers (FVF-1)* is not a core requirement. In this case, the vocabulary is still FAIR for its designed purpose within the organisation, even if the FAIR score is low. When checking the FAIR level of a vocabulary, it is important to examine the detailed use cases and features, instead of just comparing scores. A vocabulary being “FAIR enough” for its purpose is more important than having a general FAIR score. Moreover, each assessment system might have different FAIR scores for the same vocabulary. Instead of aiming for an absolute higher score, assessors should understand the mechanism behind each indicator, and focus on the interpretation of each test.

These FVF and assessments provide insights on how to improve vocabularies. For example, based on the EFO assessments, the FAIR level of EFO could easily be improved by adding a description of the aim and function of EFO, allowing different vocabulary management services to harvest that information. They also assist and guide the evolution of FAIR vocabularies by striving to iteratively improve FAIR levels of subsequently developed versions. For example, compared to ICD-10, its successor ICD-11 has incorporated many features to make it FAIRer, such as providing application programming interfaces (APIs) for easier access, having a machine-readable license, etc.

## Conclusions

We defined a FAIR vocabulary and proposed a set of features of a FAIR vocabulary. This explicitly links previous ontology standardisation efforts with the works of the fast-growing FAIR data community. The features not only cover ontology-type vocabularies but also apply to other formats of vocabulary. Furthermore, we provided a way to measure the FAIR level of a vocabulary quantitatively by aligning existing FAIR indicators with such features. This provides a foundation for further vocabulary assessment work. Finally, the features were tested against common vocabularies, and examples of how to perform FAIR assessments on vocabularies are provided. We aim to integrate existing guidelines in both the FAIR and the ontology community to deliver a comprehensive and quantitative measure of the FAIR level of vocabulary. In the future, we can develop automated tests based on the FVF requirements, perform health checks on ontology repositories and improve the ontology standards development accordingly.

## Materials and methods

### Existing vocabulary standards

Instead of reinventing a new set of features, we reused the outcomes of previous vocabulary standardisation work to determine features of FAIR vocabulary. The standards include both generic standards, such as the OBO principles [26] which cover multiple areas in ontology design and ontology development. The OBO principles aim to coordinate specifically the development of biomedical ontologies. Fourteen principles cover ontology development, ontology design and coverage. Standards targeting specific aspects of vocabularies, such as MIREOT, which focuses on ontology cross-referencing, and the OBI minimal list of metadata for term annotation, are also included. We evaluated the suitability of using such standards in the FVF definition by analysing the context and their application in discussions.

### FVF in the OLS repository

We fetched ontologies indexed in the OLS repository and selected those that are successfully loaded and up-to-date. OLS contains 266 biomedical ontologies by the time we access the database (https://www.ebi.ac.uk/ols/api/ontologies). We filtered out ontologies which could not be indexed automatically (without a valid loaded timestamp) and removed inactive ontologies based on the date information in the versionIRI section. 200 ontologies were selected based on these criteria. The loading timestamp, identifier, and version information in each ontology were checked.

We filtered out some inactive ontologies based on the loading time (only ontologies with a loading timestamp after 2019-01-01 were chosen) and date information in the version IRI (ontologies with a date before 2019-01-01 in the verionIRI field were removed). The information was collected based on machine-readable metadata imported from OLS instead of each ontology itself. But these criteria do not ensure all vocabularies selected are up-to-date. For example, for ontologies using semantic versioning format where no date information is provided in the versionIRI, or some update information is collected in other metadata fields such as ‘annotation editor comments’, etc. Despite the constraints of the analysis, it still provides enough information to showcase the status of current vocabularies.

### Development of indicators for FAIR vocabulary features

The mapping between the RDA indicators was based on text analysis, using the RDA indicator definition, description and examples. It is worth noting that when mapping the RDA indicators to datasets, *metadata* refers to the metadata to which the vocabulary can be applied, while in the context of vocabularies, *metadata* and *data* refer to the description of the vocabulary and the vocabulary information. Therefore, we combined the indicators evaluating data and metadata in the mapping we performed, wherever possible.

### Representative vocabularies

We selected three representative vocabularies to test the applicability of FVF indicators in different types of vocabularies.

ICD-11 is a large taxonomy of diseases and is the global standard for diagnostic information, disease definitions and synonyms. As a World Health Organisation(WHO) standard, ICD-11 is one of the most widely adopted disease vocabularies. It represents types of vocabularies that have low semantic maturity and is expressed as a list or a dictionary.

GO [36] is a well-established and highly regarded and utilised biomedical ontology. It contains over 43000 terms and has been cross-referenced in other classification systems, such as UniProt [37], HAMAP [38], and InterPro [39]. GO is also a reference OBO Foundry ontology [40] and has been reused in many other resources. It is selected as a representative of domain ontology.

EFO [41], on the other hand, is an application ontology built for communities like Open Targets [42] for describing experimental variables. Application ontologies, although using the standard ontology format, are mainly developed for project-specific use cases.

### Assessment against indicators for FAIR vocabulary features

We tested the FVF and corresponding indicators on three representative vocabularies, GO, EFO and ICD-11. For each FVF, three compliance levels we re-assigned; if a vocabulary meets the requirements of all indicators, *full compliance* is achieved. Otherwise, depending on the scoring for each FVF, *partial compliance* or *no compliance* results are given. The percentages of *full compliance*, *partial compliance* and *no compliance* features are also calculated. Supplementary table 3-5 provides the assessment details.

## Supplementary Information

**Additional file 1: Supplementary Table 1.** The suitability of OBO principles used as FAIR Vocabulary Features. A detailed evaluation of how suitable each OBO principle is for use as features for FAIR vocabularies.

**Additional file 2: Supplementary Table 2.** Excluded RDA data maturity indicators. RDA indicators that are not mapped to FAIR Vocabulary Features.

**Additional file 3: Supplementary Table 3.** FAIR assessment results of Gene ontology. Details of the FAIR assessment for Gene Ontology.

**Additional file 4: Supplementary Table 4.** FAIR assessment results of Experimental Factor Ontology. Details of the FAIR assessment for Experimental Factor Ontology.

**Additional file 5: Supplementary Table 5.** FAIR assessment results of ICD-11. Details of the FAIR assessment for International Classification of Diseases 11th Revision.

## Data Availability

The datasets used and analysed during this study are included in this published article and its supplementary information files.

## Abbreviations

API: Application programming interface
CC-BY: Creative commons attribution
ChEBI: Chemical entities of biological interest
EFO: Experimental factor ontology
EMBL-EBI: European molecular biology laboratory’s European bioinformatics institute
FAIR: Findable, accessible, interoperable, reusable
FTP: File transfer protocol
FVF: FAIR vocabulary features
GO: Gene ontology
HAMAP: High-quality automated and manual annotation of proteins
HTTP: Hypertext transfer protocol
HTTPS: Hypertext transfer protocol secure
ICD-10: International classification of diseases 10th revision
ICD-11: International classification of diseases 11th revision
IRI: Internationalized resource identifier
MIAPPE: Minimum information about a plant phenotyping experiment
MIBBI: Minimum information for biological and biomedical investigations
MIT license: Massachusetts institute of technology license
MIREOT: Minimum information to reference an external ontology term
NCBI: National center for biotechnology information
OBO: Open biological and biomedical ontology
OLS: Ontology lookup service
OWL: The Web ontology language
PPEO: Plant phenotype experiment ontology
PURL: Persistent uniform resource locator
RDA: Research data alliance
RDF: Resource description framework
RO: Relationship ontology
SNOMED: Systematized nomenclature of medicine
SNOMED-CT: Systematized nomenclature of medicine – clinical terms
UBERON: Uber anatomy ontology
UniProt: The universal protein resource
URI: Uniform resource identifier
WHO: World health organisation
XML: Extensible markup language
